# Synthesis and Characterization of Hydrogel Droplets Containing Magnetic Nano Particles, in a Microfluidic Flow-Focusing Chip

**DOI:** 10.3390/gels9060501

**Published:** 2023-06-19

**Authors:** Fereshteh Moharramzadeh, Seyyed Ali Seyyed Ebrahimi, Vahid Zarghami, Zahra Lalegani, Bejan Hamawandi

**Affiliations:** 1 Advanced Magnetic Materials Research Center, School of Metallurgy and Materials, University of Tehran, Tehran 11155 4563, Iranz.lalegani@ut.ac.ir (Z.L.); 2 Department of Materials and Metallurgy, Faculty of Mechanical and Energy Engineering, Shahid Beheshti University, Tehran 16589 53571, Iran; v_zarghami@sbu.ac.ir; 3 Department of Applied Physics, KTH Royal Institute of Technology, SE-106 91 Stockholm, Sweden

**Keywords:** microfluidic systems, droplet, flow-focusing system, alginate, magnetic hydrogel, magnetite

## Abstract

Magnetic hybrid hydrogels have exhibited remarkable efficacy in various areas, particularly in the biomedical sciences, where these inventive substances exhibit intriguing prospects for controlled drug delivery, tissue engineering, magnetic separation, MRI contrast agents, hyperthermia, and thermal ablation. Additionally, droplet-based microfluidic technology enables the fabrication of microgels possessing monodisperse characteristics and controlled morphological shapes. Here, alginate microgels containing citrated magnetic nanoparticles (MNPs) were produced by a microfluidic flow-focusing system. Superparamagnetic magnetite nanoparticles with an average size of 29.1 ± 2.5 nm and saturation magnetization of 66.92 emu/g were synthesized via the co-precipitation method. The hydrodynamic size of MNPs was changed from 142 nm to 826.7 nm after the citrate group’s attachment led to an increase in dispersion and the stability of the aqueous phase. A microfluidic flow-focusing chip was designed, and the mold was 3D printed by stereo lithographic technology. Depending on inlet fluid rates, monodisperse and polydisperse microgels in the range of 20–120 μm were produced. Different conditions of droplet generation in the microfluidic device (break-up) were discussed considering the model of rate-of-flow-controlled-breakup (squeezing). Practically, this study indicates guidelines for generating droplets with a predetermined size and polydispersity from liquids with well-defined macroscopic properties, utilizing a microfluidic flow-focusing device (MFFD). Fourier transform infrared spectrometer (FT-IR) results indicated a chemical attachment of citrate groups on MNPs and the existence of MNPs in the hydrogels. Magnetic hydrogel proliferation assay after 72 h showed a better rate of cell growth in comparison to the control group (*p* = 0.042).

## 1. Introduction

Hydrogels are commonly used in biomedical engineering due to their hydrated nature and adjustable properties (biocompatibility, chemical, and mechanical characteristics) close to the natural extracellular matrix (ECM) [1]. Nonetheless, there are still problems when it comes to the use of hydrogels in fields such as 3D tissue construction engineering and active targeting in drug delivery given the lack of controllability, actuation, and rapid response properties [2].

Recently, a wide range of applications and active properties of magnetic hydrogels have introduced them as a novel biocomposite. These kinds of hydrogels are utilized in tissue engineering [3], image enhancement [4], sorting and separation [5], immunoassays [6], drug delivery and release [7], immobilization of enzymes [8], cancer treatment by chemotherapy and hyperthermia [9], and soft actuation [10].

Knowing that cells exposed to magnetic fields or materials with magnetic properties can regulate several biological responses, Filippi et al. aimed to create an advanced bone regeneration platform using magnetic actuation to prime stromal vascular fraction (SVF) cells. Magnetized hydrogels, created from the co-assembly of cells, polyethylene glycol (PEG), and PEG-coated superparamagnetic iron oxide NPs, were used to enhance SVF biological function under an external magnetic field [11].

Furthermore, anisotropic tissue patterns in bioengineered constructs are challenging. Using responsive magnetic hydrogels for 3D bioprinting of scaffolds and magneto-mechanical actuation can create an ideal strategy for anisotropic mechanosensitive tissue engineering [12].

Magnetic targeting enables the targeting of a tumor and hence prevents the systematic distribution of cytotoxic compounds; this leads to an enhancement of drug uptake at the target site inside the body and lowers the required doses for effective treatment. Furthermore, it was shown that magnetic hydrogels along with the alternating magnetic field could be used as hyperthermia treatment by inducing tumor regression [13,14].

In practice, micron-sized and morphologically homogeneous monodisperse composite particles are preferred since they have reproducible and consistent behavior. Particles are required to be biocompatible and biodegradable to be used in biomedical applications [15].

Recently, some microfluidic devices were utilized for the fabrication of polymeric microparticles that are widely utilized in the delivery of cells and drugs, 3D-cell culture, tissue engineering, and immunoassays [13,14,16,17].

The control of the size, shape, and polydispersity along with the precise generation and repeatability of droplet operations, have made a droplet-based microfluidic system a potent high throughput platform for biomedical research and applications. In addition to being used as micro reactors, droplet-based systems have also been used to directly synthesize particles and encapsulate many biological entities for biomedicine and biotechnology applications [18,19].

The MFFD, T-junctions, and those that liquid threads break on the terraces of the microchannel which the dispersed phase, break upon their intersection with the continuous phase at the most effective area on-chip, and they pinch off into micron-sized droplets which are subsequently suspended in the continuous phase. These are among the widely used micro emulsification apparatus. Meanwhile, the production of droplets with an expectable and reproducible size distribution determines their possible uses (including the synthesis of polymer colloids), numerous researchers have investigated different features of the emulsification procedure. It was concluded that the controlling parameters of droplet sizes include liquids’ properties, flow rates of the two immiscible phases, and the microfluidic device design [20].

In this study, the dynamics of the break-up were discussed considering the model of rate-of-flow-controlled-breakup (or squeezing). According to this model, the droplet size was dependent on the flow rate ratio of the two dispersed and continuous phases. Practically, our study indicates guidelines for generating droplets with a predetermined size and polydispersity from liquids with well-defined macroscopic properties, utilizing a MFFD. Additionally, significant biocompatibility and cell proliferation of alginate hydrogels containing citrated magnetic NPs present the high potential of these synthetic materials for novel applications such as 3D bioprinting of intrinsic magnetic scaffolds, injectable granulated hydrogels, and synergistic and targeted cancer therapy using controlled drug delivery and hyperthermia. This study provides a useful approach and comprehensive guideline for obtaining complex biocompatible materials with active magnetic properties.

## 2. Results and Discussion

### 2.1. Properties of Magnetic NPs Synthesized by Co-Precipitation Method

#### 2.1.1. Morphology and Particle Size

**Figure 1:**
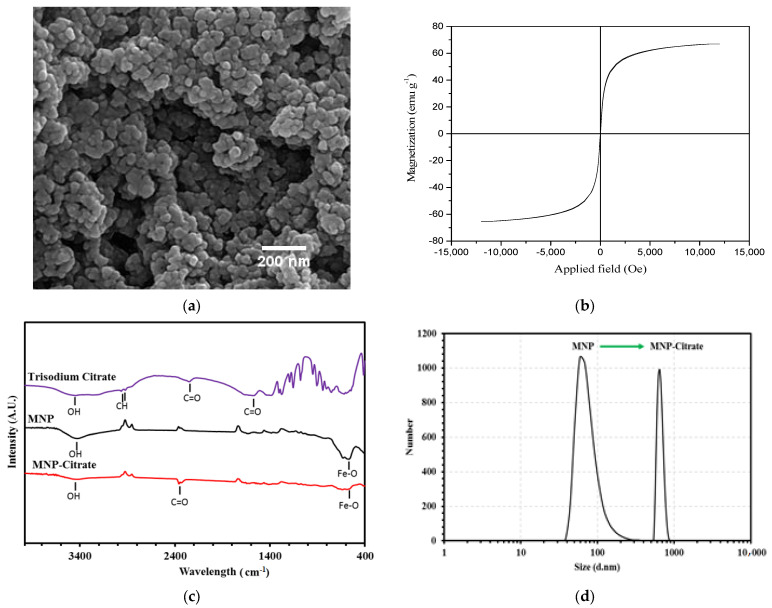
(**a**) FE-SEM image of magnetite NPs synthesized by the co-precipitation method, (**b**) M-H diagram of magnetite NPs synthesized by the co-precipitation method, (**c**) FT-IR spectra of trisodium citrate, magnetite NPs, and citrated magnetite NPs, and (**d**) hydrodynamic size of magnetite NPs before and after citration.

The FE-SEM image of magnetite NPs is shown in Figure 1a. According to Figure 1a, the morphology of the synthesized magnetite NPs was spherical with the mean as-prepared nanoparticle size of 29.1 ± 2.5 nm. The average particle size was obtained after measuring the diameter of 50 NPs in the image.

#### 2.1.2. Magnetic Properties

Magnetic measurements of NPs were performed using a vibrating sample magnetometer (VSM, MagKav Co., Kashan, Iran) at room temperature. One of the special properties of magnetic NPs is their superparamagnetic behavior. When a magnetic material dimension is in nanoscale, each magnetic domain is located in a separate NP. When the particle size is less than 30 nm, they inhibit superparamagnetic behavior [21]. The VSM curve of synthesized NPs at room temperature is shown in Figure 1b. As seen in Figure 1b, the coercivity and remanence are zero. In addition, the saturation magnetization is 66.92 emu g^−1^.

#### 2.1.3. FT-IR Investigation

The FT-IR spectra of samples were obtained using a Perkin-Elmer 781 nanoparticle spectrophotometer (Waltham, MA, USA). In this study, the samples were formulated as tablets in solid KBr solution and the spectrum was obtained in the mid-range of 4000–400 cm^−1^.

The FT-IR of bare magnetic NPs, citrated magnetic NPs, and trisodium citrate as the control group are shown in Figure 1c. Two main peaks of magnetite in the FT-IR spectrum have appeared in the wavelength range of 550–650 cm^−1^ showing Fe–O groups. The peak at 3450 cm^−1^ is due to the stretching vibrations of O-H groups [22]. According to the FT-IR spectrum of citrated NPs, there is a new peak at 2352 cm^−1^. The corresponding peak in the spectrum of trisodium citrate is 2250 cm^−1^. This indicates that the citrate is attached to the oxygen groups on the magnetite via the covalent bond and resulted in the C=O bond’s shift from 2250 to 2352 cm^−1^. This shift indicates the formation of a carboxyl bond between sodium citrate and magnetite NPs [23].

#### 2.1.4. Hydrodynamic Size Investigation

The hydrodynamic size distributions of NPs were taken using dynamic light scattering (DLS, Malvern Zetasizer Nano ZS, Malvern, UK).

The DLS results of NPs before and after being citrated are shown in Figure 1d. The hydrodynamic size before being citrated was 142 nm which reached 826.7 nm after being citrated. Being citrated causes an increase of NP stability in the solution and prevents sedimentation during the microfluidic process.

### 2.2. Micro Gel Production in the Microfluidic Chip

In this research, experiments were performed based on the flow-focusing geometry for the formation of droplets in a continuous phase (oil phase) from an immiscible phase (hydrogel precursors). This method enables us to produce high-speed and one-step hydrogels’ production in the desired dimensions. The microfluidic chip efficiency in microgels’ synthesis was imaged by a biological optical microscope (CX22 Olympus, Tokyo, Japan) which was equipped with a recorder camera system.

**Figure 2:**
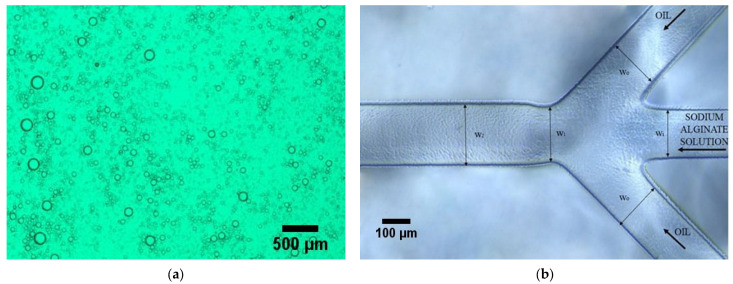
(**a**) Microscopic image of collected microgels produced in the spinal chip, (**b**) Optical microscope image of triple junction in microfluidic chip.

Two different designs of microfluidic chips were used; the triple junction geometry was the same for both designs. Droplet formation was investigated in both designs and droplet formation in the triple junction in both chips was in the same condition of flow rate ratio. In the chip with spiral channels, the size of collected droplets was not uniform because the spiral design acts as a mixer, and in the channel path, they mix to etch other or separate into smaller droplets (Figure 2a). To find the average size of hydrogels in an image, approximately 100 hydrogels were measured and averaged by ImageJ software v1.53. The average diameter of microgels is 38.7 (±10) μm. Droplets produced in the chips with parallel channels approximately were not mixed or separated. They were collected at the triple junction point. Hence, in this study, the results of the droplets produced in the chip with parallel channels were reported.

A wide range of droplet formation patterns were observed based on the input rates of two phases. The droplets’ size change was quantified by two parameters: the oil phase rate (Q_o_) and the ratio of the hydrophilic phase input rate to the oil phase rate (Q_i_/Q_o_). The oil phase rate has assumed as the sum of the input rates of two oil channels. The formation of droplets was observed both uniformly and non-uniformly. The geometry of the utilized microfluidic system is shown in Figure 2b, where w_o_ is 160 μm, w_i_ and w_1_ are 110 μm, and w_2_ is 200 μm. The oil phase flows from the two side channels and the hydrophilic phase flows from the middle channel. A shear force is applied from the two oil flows to the hydrophilic phase and disrupts it, and the droplets are formed.

In this research, the input rate of the oil phase (Q_o_) was always greater than the input rate of the hydrophilic phase (Q_i_). Two ratios of Q_i_/Q_o_ = 1/5 and 1/20 were selected and studied. The oil phase rate has assumed as the sum of the input rates of two oil channels and was tuned from 0.2 to 20 mL h^−1^. Droplet formation was observed and recorded by the optical microscope and the connected camera.

After the completion of numerous experiments for the formation of droplets in the microfluidic system, the following table was achieved for various cases.

**Figure 3:**
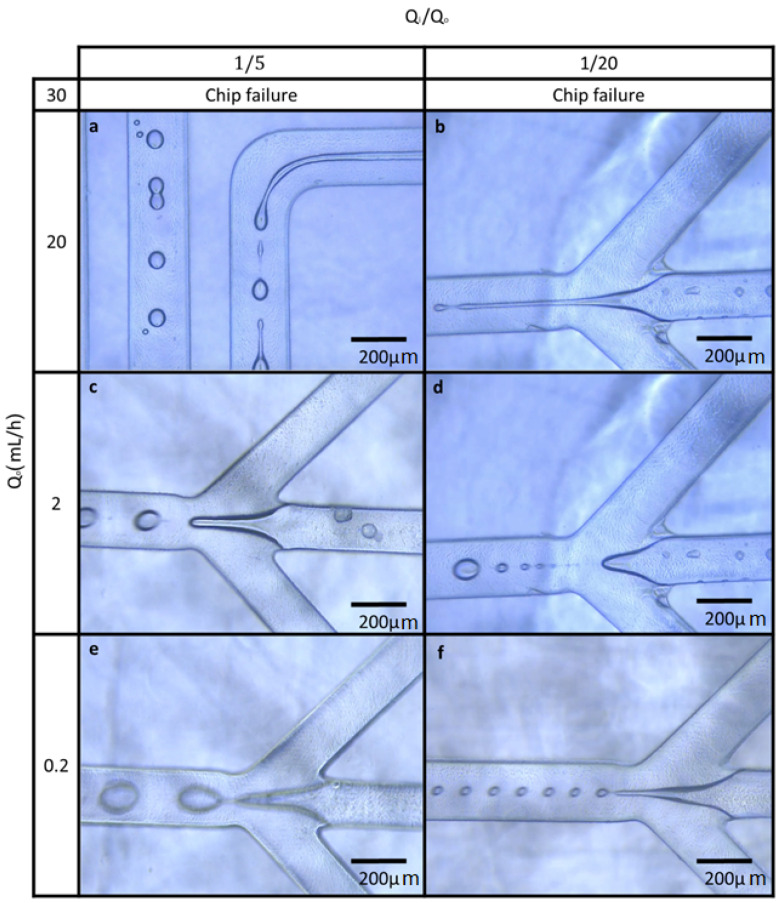
Different modes of droplet production in the microfluidic system, regarding the physical properties of phases that are constant in this study jetting mode (**a**,**b**), dripping mode (**c**,**e**,**f**) and satellite formation (**d**) were observed.

Figure 3 shows different modes of droplet production in the microfluidic system. According to Figure 3, in the case of Q_o_ = 2 mL h^−1^ and Q_i_/Q_o_ = 1/20, the formation of droplets is along with the formation of some satellites (Figure 3d), which indicates a non-uniform distribution of droplets’ sizes. In the case of Q_o_ = 0.2 mL h^−1^ and Q_i_/Q_o_ = 1/20, the formation of droplets with more homogeneity in size distribution can be seen (Figure 3f). The droplet separation occurs in the place of channel narrowing.

According to Figure 3c,e,f, a uniform size distribution of the droplets can be observed. Furthermore, in some cases (Q_o_ = 20 mL h^−1^ and Q_i_/Q_o_ = 1/5) the droplets merged and formed larger droplets when they were crossing through the channels and subsequently, in the pathway (Figure 3a). Regarding the physical properties of phases that are constant in this study, two different modes occurred: the dripping and jetting created by changing the two phases’ flow rates. In the case of dripping, the middle phase flow is interrupted in the narrowing place of the channel or near it and the droplets are formed. (Figure 3c,e,f). In the jetting mode, the middle phase extends several hundreds of micrometers downstream of the narrowing section, and may (droplet formation in Figure 3a,b) or may not (fiber formation) be interrupted.

With constant Q_i_/Q_o_ and small values of Q_i_ and Q_o_ (0.2 mL h^−1^), the dripping mode occurs and larger droplets are formed (Figure 3e with a diameter of about 120 μm).

In moderate values of Q_i_ and Q_o_ (2 mL h^−1^), the dripping mode occurs again; the droplet size distribution is more uniform and smaller droplets are formed rather than low velocities (Figure 3c with a diameter of about 75 μm). In a narrow range of moderate values of Q_i_ and Q_o_, small satellite droplets were formed with a non-uniform size distribution (Figure 3a).

At high values of Q_i_ and Q_o_ (20 mL h^−1^), the jet stream occurs and the middle phase extends several hundreds of micrometers downstream of the narrowing section (Figure 3a,b). In tests carried out during the transition between the dripping and jetting mode, the tip of the middle phase extends abruptly and was unstable.

The droplets produced in this case were initially fine but they merged with each other and larger droplets formed, so the size distribution became non-uniform [20]. In Figure 3a, the coagulation of droplets in the channel can be seen.

### 2.3. Characterization of Microgels Containing Magnetic NPs

Microscopic images of hydrogels were obtained by fluorescence microscope (Olympus BX51, Japan) in transmission mode without any fluorescent labeling in order to ensure their structures and morphologies were not disrupted. The size of about 100 hydrogels in each photo was measured by ImageJ software and averaged to obtain hydrogel average diameters.

**Figure 4:**
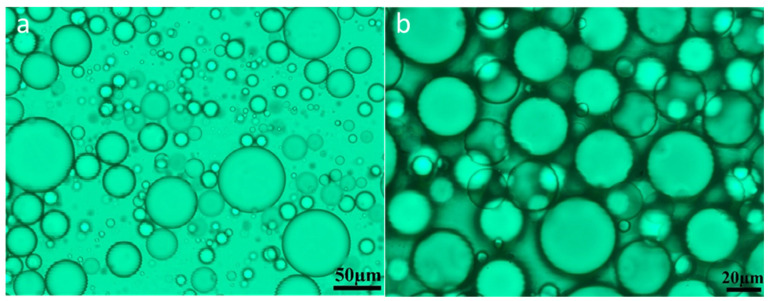
Microscopy image (in transmission mode) of: (**a**) non-uniform distribution of droplet sizes and (**b**) near uniform distribution of droplet sizes.

Figure 4a shows microgels with non-uniform sizes with an average diameter of 25.64 μm resulting from the middle-phase jet flow. In Figure 4b, where the middle phase flow rate is reduced, the average diameter of the microgels is reduced to 19.5 μm and the size distribution is nearly uniform. The heterogeneity in droplet sizes was analyzed by a developed model by Higgins et al. [24]. According to their model, the effect of heterogeneity was quantified by the degree of inconsistency in the studies’ results (I^2^ value, the percentage of observed total variation across studies, which is due to real heterogeneity rather than chance). The heterogeneity in the size of microgels in Figure 4a was calculated at 71%, while it was 21% for microgels in Figure 4b; knowing that the value of 0% indicates no observed heterogeneity and larger values show increasing heterogeneity.

**Figure 5:**
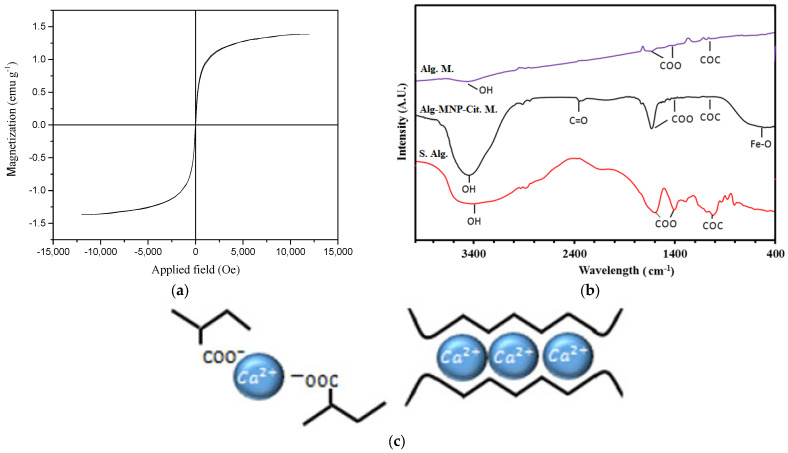
(**a**) M-H diagram of alginate microgels containing magnetite NPs, (**b**) FT-IR spectra of sodium alginate (S.Alg), alginate microgels (Alg.M.) and alginate microgels containing citrated magnetite NPs (Alg.MNP-Cit.M), and (**c**) ionic bonding interaction between Ca^2+^ and carboxylate groups in alginate hydrogels.

Figure 5a shows the VSM result of alginate microgels containing magnetite NPs. According to Figure 5a, coercivity and remanence are zero, therefore the encapsulated NPs in hydrogels preserve the superparamagnetic property. The saturation magnetization is 1.38 emu g^−1^. The reduction of saturation magnetization in hydrogels containing magnetic NPs (in comparison with magnetite) can be due to non-crystalline structures’ attachment on magnetic NPs. Any crystalline disorder in the surface of NPs can increase surface anisotropy and significantly reduce saturation magnetization [25]. In addition, the weight percentage of MNPs in microgels containing magnetic NPs decreases, and the diamagnetic hydrogel surrounds the Fe_3_O_4_; both lead to a decrease in magnetic properties [26].

The FT-IR spectrum of alginate hydrogels with and without magnetite NPs is shown in Figure 5b. In order to have a better comparison, the spectrum of sodium alginate is also taken. In the sodium alginate spectrum, the main peaks that appeared at 3374, 1610, 1415, and 1036 cm^−1^ were related to OH, COO (asymmetric stretching vibrations), COO (symmetric stretching vibration), and COC bonds, respectively.

These peaks in the alginate microgels spectrum appeared at 3390, 1600, 1415, and 1037 cm^−1^ for OH, COO (asymmetric stretching vibrations), COO (symmetric stretching vibration), and COC bonds, respectively [27,28]. There is a small shift in peaks’ locations in microgels. The reason for this shift can be due to the structure folding of the molecules and ionic bonds’ formation between carboxylate groups and calcium ions. Furthermore, no new peak formation indicates no new covalent bond formation between the comprising hydrogel molecules. Figure 5c schematically describes the mechanism of the egg box structure formation and ionic cross-linking of the alginate chains. Egg box structure refers to the formation of divalent metal ions, such as calcium or magnesium, cross-linking with carboxylate groups in alginate. This results in a 3D network of chains that provides stability to the alginate matrix [29]. The strength of hydrogels could be ascribed to the crosslinking ability of calcium ions. D’Elía et al. [30] explained that hydrogels with greater levels of Ca^2+^ tend to have higher storage modulus (energy stored in the elastic structure) values in the linear viscoelastic range which results in higher strength. Moreover, this structure reduces the water content within the gel, which contributes to its stability by limiting the mobility of the polymer chains and preventing the chains from becoming soluble in an aqueous solution [31].

In the alginate microgels and citrated magnetite NPs spectrums, the formation of a bond between citrate and magnetite NPs (C=O) appeared at 2356 cm^−1^ [23,32].

The slight shift of this peak also indicates the weak bonds between alginate and citrate on the surface of NPs. The peaks appeared at 570, 1415, 1612, 1037, and 3405 cm^−1^ which are due to Fe-O, COO (symmetric stretching vibrations), COO (asymmetric stretching vibration), COC, and OH, respectively [33,34,35]. This result proves the presence of magnetite NPs inside the hydrogels.

### 2.4. Biocompatibility of Magnetite NPs before and after Citration

**Figure 6:**
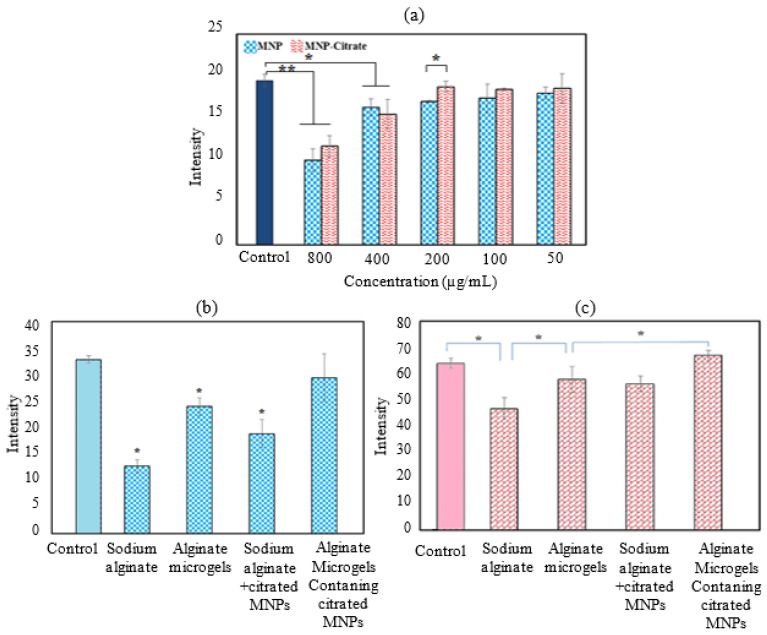
(**a**) Magnetite NPs biocompatibility before and after citration at different concentrations in the presence of human mesenchymal stem cells after 24 h (*: *p* < 0.05, **: *p* < 0.001); (**b**) biocompatibility test of alginate based microgels and their constituents after 1 day (*: *p* < 0.05 in comparison with control group); and (**c**) biocompatibility test of alginate based microgels and their constituents after 3 days. (*: *p* < 0.05).

The cytotoxicity results of magnetite NPs before and after citration at different concentrations are shown in Figure 6a. The significance of differences between the biocompatibility of magnetite NPs and citrated magnetite NPs at different concentrations within the control group was statistically analyzed by SPSS software. The group statistics of the measurement results were analyzed using ANOVA post-hoc Tukey. The value of *p* < 0.05 was defined as a condition of significant differences. If the significant difference parameter (Sig) is less than 0.05, the difference is significant and means a decrease in cell growth compared to the control group. At 200 μg μL^−1^ and lower concentrations, the cytotoxicity of both bare magnetite NPs and citrated magnetite NPs were not significant. However, in the citrated NPs, at the concentration of 200 μg μL^−1^, the rate of stem cell growth was significantly higher than similar concentrations of non-citrated NPs (*p* = 0.009634). Proper cell growth in the presence of citrated magnetite NPs indicates a decrease in cytotoxicity and an increase in their biocompatibility through the citration process.

Cellular biocompatibility resulting from microgels (with and without magnetite NPs) and their constituents prior to gelation for 24 and 72 h of cell proliferation is shown in Figure 6b,c. According to statistical analysis by SPSS v29 software, the cell growth rate in alginate microgels compared to alginate solution after 24 h of cell culture has a significant parameter of 0.002. Given this low *p* value, gelation has increased the bioactivity of the initial solutions. Similar to hydrogels without citrated magnetite NPs, there are similar conditions in microgels containing NPs before and after gelation. Cell proliferation in alginate microgels containing NPs during the first 24 h of culture was significantly more than that of the hydrogels’ precursor.

After one day only, alginate nanoparticles containing citrated magnetite nanoparticles had no significant difference with the control group and there was no decrease in cell growth and proliferation in this group.

The cell growth rate in alginate microgels compared to alginate solution after 72 h of cell culture has a significant parameter of 0.017, then sodium alginate microgels have better cell proliferation than sodium alginate. Cell proliferation in alginate microgels containing citrated magnetite NPs was significantly higher than net alginate microgels within 72 h post-culture (*p* = 0.042). It could be due to how citrate groups (Ca^2+^ chelator) induce a structural switch in a tight alginate network and produce a more permissive microenvironment with a more open network [36]. This type of switch affects not only the structural features (i.e., the mesh size) but also the mechanical properties of hydrogels. In addition, diffusion of biological compounds and cell motility may be affected by alteration in the network mesh size [37].

The significant difference in cell proliferation in all groups after 72 h was compared by pairwise comparison. After three days, all groups except for the sodium alginate group had no significant difference and were able to have good cell proliferation.

## 3. Conclusions

In this study, alginate microgels containing citrated magnetite NPs were produced by the microfluidic flow-focusing device. Superparamagnetic NPs with an average size of 29.1 ± 2.5 nm and saturation magnetization of 66.92 emu g^−1^ were synthesized via the co-precipitation method. The citration process was performed on NPs in order to prevent MNPs’ agglomeration. DLS results indicated an increase in MNPs hydrodynamic diameter from 142 nm to 826.7 nm after citration so dispersion and stability in the aqueous phase was improved. In addition, cell culture analysis showed significantly higher biocompatibility of citrated MNPs in comparison with non-citrated MNPs in the concentration of 200 μg μL^−1^. The alginate droplets were produced in the MFFD and the size and polydispersity of droplets were discussed in relation to the flow rate ratio of two immiscible phases of oil and hydrogel precursor based on the model of rate-of-flow-controlled-breakup (or squeezing). FT-IR results stated the chemical attachment of citrate groups on MNPs and the existence of MNPs in the hydrogels. The values of coercivity and remanence remained at zero after encapsulation of citrated MNPs in alginate hydrogels but the saturation magnetization decreased to 1.38 emu g^−1^ due to non-crystalline structures attachment on magnetic NPs. Cell proliferation in alginate microgels containing citrated magnetite NPs was significantly higher than net alginate microgels within 72 h post-culture (*p* = 0.042). Moreover, at 200 μg μL^−1^ and lower concentrations, the cytotoxicity of magnetite NPs and citrated magnetite NPs were not significant. In brief, the results indicated that alginate hydrogels containing citrated MNPs produced in this work showed significant biocompatibility. On the other hand, preserving the superparamagnetic properties of MNPs after trapping them in hydrogel makes this multifunctional material suitable for magnetic resonance imaging (MRI), hyperthermia, magnetic active targeted drug delivery, tissue engineering scaffolds, and 3D cell culture applications. Furthermore, the predetermined size and polydispersity with well-defined macroscopic properties, utilizing a MFFD achieved in this work, would be an important step to progress using injectable granulated hydrogels in regenerative medicine.

## 4. Materials and Methods

### 4.1. Materials

Poly(dimethylsiloxane) and SYLGARD184 silicone elastomer kit were from Dow Corning. Soybean oil, sodium alginate, calcium acetate, sodium citrate (citric acid trisodium salt dehydrate), span 80, resazurin sodium salt, phosphate-buffered saline (PBS), penicillin, and streptomycin were purchased from Sigma-Aldrich company (Washington, DC, USA).

FeCl_3_, FeCl_2_.4H_2_O, and NH_4_OH were purchased from Merck company (Darmstadt, Germany). Minimum essential medium alpha (MEM α) and trypan blue solution 0.4% were bought from Fisher Scientific (Portsmouth, NH, USA). Fetal bovine serum (FBS) and trypsin were purchased from Life Technologies company (Carlsbad, CA, USA). More details on materials specifications are listed in Table 1.

### 4.2. Methods

#### 4.2.1. Magnetite Nanoparticles Synthesis

The co-precipitation process was used for the synthesis of Fe_3_O_4_ magnetic NPs. For this purpose, 150 mL of deionized water containing 78 mM of FeCl_3_ and 150 mL of deionized water containing 39 mM of FeCl_2_.4H_2_O were prepared separately. The solutions were magnetically stirred and then mixed in a tree neck balloon under the nitrogen atmosphere. The solution was then heated up to 80 °C and 10 mL of 25% NH_4_OH was added to the stirring mixture dropwise until pH reached 11. The precipitates were then separated by the centrifuge and washed one time with the water and centrifuged again to remove the reaction by-products and finally were dried at room temperature.

#### 4.2.2. 3D-Printing of the Mold

**Figure 7:**
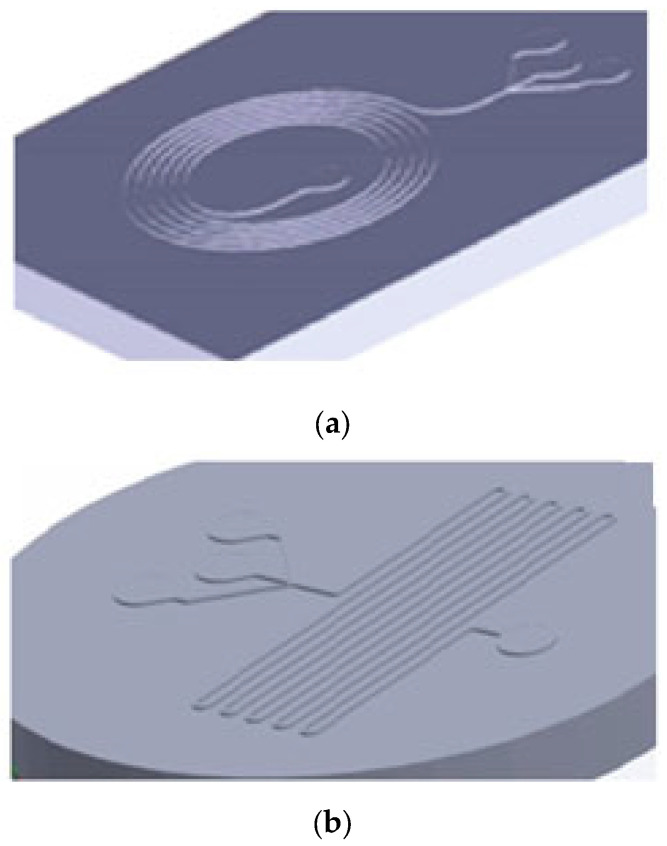
3D view of designed models for microfluidic chip mold with: (**a**) spinal channel and (**b**) parallel channels.

Two different designs of molds (Figure 7) were printed by EnvisionTEC with the dimension accuracy of 25 μm by 84 mm lens and PIC 100 resin. The prepared models were washed with ethanol and then immersed in an ethanol bath for 20 min to remove the remaining resin from the surface. After drying, the models were exposed to a visible light flasher to cure the resin completely to prevent any crack formation in the next step when molding by PDMS in the oven. The samples were exposed to 1000 flashes. For this purpose, a flasher device with the frequency of 1 flash per second was used. Finally, the samples were washed with ethanol and deionized water in order to remove any more pollution from the surface of the samples.

#### 4.2.3. Synthesize of Citrated Magnetite Nanoparticles 

**Figure 8:**
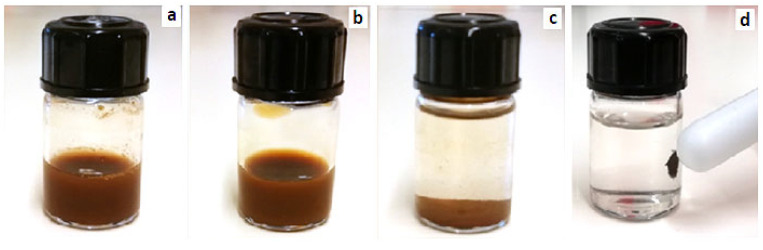
Citration steps of Fe_3_O_4_ NPs: (**a**) after the addition of deionized water to the magnetic NPs and dispersion in an ultrasonic bath, (**b**) after the addition of sodium citrate and placing it in the oven, (**c**) after the addition of acetone and precipitation of citrated magnetic NPs, and (**d**) the attraction of citrated magnetic NPs to the magnet.

For this purpose, 1.5 mg of synthesized magnetite NPs were dispersed in 2 mL of deionized water in an ultrasonic bath for 2 h (Figure 8a). Then, 1.5 mg of sodium citrate was added to the mixture and heated to 80 °C for 60 min (Figure 8b). Immediately after removing the mixture from the oven, 2 mL of acetone were added and the resultant mixture was stored at room temperature overnight. During this time, the citrated NPs were precipitated and the upper solution was separable (Figure 8c,d). After sucking the top solution, the sediments were separated.

#### 4.2.4. Production of Microfluidic Chip

PDMS and the curing agent were mixed in at a ratio of 1:10 wt% in order of the microfluidic chip molding and then placed in the vacuum chamber for 30 min to be degassed. The vacuum was removed and applied 2–3 times. The models remained for 45 min in a 75 °C oven for PDMS curing. In the next step, the punched PDMS replica and bare PDMS plate surface were activated by plasma spray and coupled together. The chips remained for 12 h in a 115 °C oven to achieve a stronger bond.

#### 4.2.5. Solutions Preparation

A total of two hydrophilic and hydrophobic phases were prepared for the synthesis of microgel droplets in the chip.

To prepare for the hydrophilic dispersed phase, 1 wt% of sodium alginate aqueous solution was prepared by adding a certain amount of sodium alginate salt to DI water and it was maintained for 2 h in an incubator on rotating axels. The solution passed through a 450 nm filter to remove any unsolved particles and dust. A total of 500 μL of deionized water were added to 1.5 mg of citrated magnetite NPs and pipetaged for several times. The solution was added to 2 mL of a 1 wt% of sodium alginate aqueous solution and mixed using a vortex mixer.

To prepare for the hydrophobic continuous phase, a solution containing 2 wt% of calcium acetate and 3 wt% of Span 80 surfactant in soybean oil were prepared and vortexed for 30 min and then mixed for 12 h by magnetic stirring. The organic solution was also filtered by a 450 nm filter to remove any unwanted particles. A collection reservoir solution containing 3 wt% of Span 80 surfactant in soybean oil was prepared and magnetically stirred for 30 min. This solution was used for saving the hydrogel droplets outgoing from the microfluidic chip.

#### 4.2.6. Droplet Formation

Different input rates were tested to investigate the possibility of the formation of a stable droplet in the chip. The examined rate ranges (sum of two rates from two channels) were 30–0.2 mL and 3–0.01 mL per hour for the hydrophobic and hydrophilic phases, respectively. It should be noted that flow rates higher than 30 mL h^−1^ resulted in chip failure because the PDMS attachment to glass was not strong enough to overcome those flow rates. Finally, the achieved droplets were stored in 3 wt% of Span 80 in Soybean oil.

#### 4.2.7. Cell Culture

Human mesenchymal stem cells (hMSCs) were used in this study. The cell line was obtained from the national cell bank (Pasteur Institute of Iran, Tehran, Iran). The hMSCs were proliferated in α MEM medium supplemented with 10% of FBS and 1% of penicillin, and 1% of streptomycin in 125 cm^2^ flasks at 37 °C in a sterile incubator. Cell counting was performed by an automated cell counter, which was used to evaluate cytotoxicity.

#### 4.2.8. Alamar Blue Cell Proliferation Test

Due to the high potential of the produced hydrogels as an MRI contrast agent and as bio-ink for 3D printing of scaffolds and injectable hydrogels, the toxicity test was performed. For this purpose, the alamar blue method was used for a cell proliferation assay with NPs and hydrogels. The NPs and hydrogels were exposed to UV radiation for 40 min prior to cell testing to eliminate possible microbial contamination.

To investigate the toxicity or cell proliferation of NPs and hydrogels, 10,000 cells were plated on each of the 96-well plates. Approximately 200 μL of α MEM medium containing 10% of FBS and 1% of antibiotic were added to each well. Plates were incubated at 37 °C for 24 h. The amount of CO_2_ incubated in cell culture was adjusted to 5%. After one day, the cells were stuck to the bottom of the plate. The cell culture medium was evacuated and replaced with a culture medium containing a certain amount of NPs or hydrogels. To investigate the cell proliferation potential of the specimens, hydrogels and NPs were placed into the wells at the bottom of the 96-well plates, then 200 μL of culture medium containing 10,000 cells was poured onto them. Cellular proliferation was determined after a specific time by the alamar blue assay. The procedure was performed by adding 20 μL of alamar blue’s solution containing 40 mM of resazurin sodium salt in PBS medium to each well. Then it was incubated in the dark for 4 h at 37 °C. A total of 100 μL of the top solution of each well was transferred to a new plate. The absorbance of each well, indicating cell viability, was measured by the Elisa Plate Reader (Thermo Scientific, Waltham, MA, USA) at 570 nm. At the same time, the absorbance was measured at 630 nm to eliminate the effect of the background on absorption. Each process was repeated three times. In addition, wells containing cells without NPs and hydrogels were selected as the control group.

## Figures and Tables

**Table 1:** Specifications of materials used in this study.

Material	Company	CAS No.
FeCl_3_	Merck	7705-08-0
FeCl_2_.4H_2_O	Merck	13478-10-9
NH_4_OH	Merck	1336-21-6
Ethanol	Merck	64-17-5
Acetone	Merck	67-64-1
Sodium citrate	Sigma Aldrich	6132-04-3
PDMS	Sigma Aldrich	9016-00-6
Sodium alginate	Sigma Aldrich	9005-38-3
Calcium acetate	Sigma Aldrich	114460-21-8
Soybean oil	Sigma Aldrich	8001-22-7
Span 80	Sigma Aldrich	1338-43-8

## Data Availability

The data presented in this study are available on request from the first author.
